# Supplementary material for: The impact of technical efficiency on firms’ value: The case of the halal food and beverage industry in selected countries
Source: PLoS One. 2023 Nov 27;18(11):e0286629. doi: 10.1371/journal.pone.0286629 (PMC10681182; doi:10.1371/journal.pone.0286629)
Supplement: S1 File — (DOCX) [file pone.0286629.s003.docx]

[https://doi.org/10.6084/m9.figshare.21971270](https://doi.org/10.6084/m9.figshare.21971270)

# Malaysia

Chow Test

| Redundant Fixed Effects Tests | | |  |  |
| --- | --- | --- | --- | --- |
| Equation: FEM | |  |  |  |
| Test cross-section fixed effects | | | |  |
|  |  |  |  |  |
|  |  |  |  |  |
| Effects Test | | Statistic | d.f. | Prob. |
|  |  |  |  |  |
|  |  |  |  |  |
| Cross-section F | | 6.314580 | (16,63) | 0.0000 |
| Cross-section Chi-square | | 81.339438 | 16 | 0.0000 |
|  |  |  |  |  |
|  |  |  |  |  |

Hausman Test

| Correlated Random Effects - Hausman Test | | | |  |
| --- | --- | --- | --- | --- |
| Equation: REM | |  |  |  |
| Test cross-section random effects | | | |  |
|  |  |  |  |  |
|  |  |  |  |  |
| Test Summary | | Chi-Sq. Statistic | Chi-Sq. d.f. | Prob. |
|  |  |  |  |  |
|  |  |  |  |  |
| Cross-section random | | 0.000000 | 5 | 1.0000 |
|  |  |  |  |  |
|  |  |  |  |  |

**Table 7. Malaysia Panel Regression Result**

| Dependent Variable: Y_TQ | | |  |  |
| --- | --- | --- | --- | --- |
| Method: Panel EGLS (Cross-section random effects) | | | | |
| Date: 07/29/22 Time: 10:48 | | |  |  |
| Sample: 2017 2021 | | |  |  |
| Periods included: 5 | | |  |  |
| Cross-sections included: 17 | | |  |  |
| Total panel (balanced) observations: 85 | | | |  |
| Swamy and Arora estimator of component variances | | | | |
|  |  |  |  |  |
|  |  |  |  |  |
| Variable | Coefficient | Std. Error | t-Statistic | Prob. |
|  |  |  |  |  |
|  |  |  |  |  |
| C | -0.981138 | 6.637995 | -0.147806 | 0.8829 |
| X1_TE | 3.206398 | 1.399744 | 2.290703 | 0.0246 |
| X2_SIZESALES | -0.058052 | 0.365932 | -0.158641 | 0.8744 |
| X3_DAR | 10.21923 | 2.180221 | 4.687245 | 0.0000 |
| X4_GROWTH | -0.080369 | 1.543847 | -0.052057 | 0.9586 |
| X10_INF | -0.125324 | 0.134695 | -0.930424 | 0.3550 |
|  |  |  |  |  |
|  |  |  |  |  |
|  | Effects Specification | |  |  |
|  |  |  | S.D. | Rho |
|  |  |  |  |  |
|  |  |  |  |  |
| Cross-section random | | | 1.745582 | 0.4380 |
| Idiosyncratic random | | | 1.977420 | 0.5620 |
|  |  |  |  |  |
|  |  |  |  |  |
|  | Weighted Statistics | |  |  |
|  |  |  |  |  |
|  |  |  |  |  |
| R-squared | 0.232316 | Mean dependent var | | 1.349122 |
| Adjusted R-squared | 0.183728 | S.D. dependent var | | 2.413160 |
| S.E. of regression | 2.180236 | Sum squared resid | | 375.5209 |
| F-statistic | 4.781384 | Durbin-Watson stat | | 1.592158 |
| Prob(F-statistic) | 0.000720 |  |  |  |
|  |  |  |  |  |
|  |  |  |  |  |
|  | Unweighted Statistics | |  |  |
|  |  |  |  |  |
|  |  |  |  |  |
| R-squared | 0.454988 | Mean dependent var | | 2.985282 |
| Sum squared resid | 750.3113 | Durbin-Watson stat | | 0.796854 |
|  |  |  |  |  |
|  |  |  |  |  |

# Indonesia

Chow Test

| Redundant Fixed Effects Tests | | |  |  |
| --- | --- | --- | --- | --- |
| Equation: FEM | |  |  |  |
| Test cross-section fixed effects | | | |  |
|  |  |  |  |  |
|  |  |  |  |  |
| Effects Test | | Statistic | d.f. | Prob. |
|  |  |  |  |  |
|  |  |  |  |  |
| Cross-section F | | 22.104635 | (11,43) | 0.0000 |
| Cross-section Chi-square | | 113.719169 | 11 | 0.0000 |
|  |  |  |  |  |
|  |  |  |  |  |

Hausman Test

| Correlated Random Effects - Hausman Test | | | |  |
| --- | --- | --- | --- | --- |
| Equation: REM | |  |  |  |
| Test cross-section random effects | | | |  |
|  |  |  |  |  |
|  |  |  |  |  |
| Test Summary | | Chi-Sq. Statistic | Chi-Sq. d.f. | Prob. |
|  |  |  |  |  |
|  |  |  |  |  |
| Cross-section random | | 0.000000 | 5 | 1.0000 |
|  |  |  |  |  |
|  |  |  |  |  |

**Table 8. Indonesia Panel Regression Result.**

| Dependent Variable: Y_TQ | | |  |  |
| --- | --- | --- | --- | --- |
| Method: Panel EGLS (Cross-section random effects) | | | | |
| Date: 07/29/22 Time: 10:57 | | |  |  |
| Sample: 2017 2021 | | |  |  |
| Periods included: 5 | | |  |  |
| Cross-sections included: 12 | | |  |  |
| Total panel (balanced) observations: 60 | | | |  |
| Swamy and Arora estimator of component variances | | | | |
|  |  |  |  |  |
|  |  |  |  |  |
| Variable | Coefficient | Std. Error | t-Statistic | Prob. |
|  |  |  |  |  |
|  |  |  |  |  |
| C | -3.028196 | 3.723861 | -0.813187 | 0.4197 |
| X1_TE | 0.929285 | 0.346539 | 2.681620 | 0.0097 |
| X2_SIZESALES | 0.268421 | 0.186266 | 1.441066 | 0.1553 |
| X3_DAR | -3.788711 | 0.698798 | -5.421756 | 0.0000 |
| X4_GROWTH | -0.151444 | 0.253955 | -0.596343 | 0.5534 |
| X10_INF | 0.195911 | 0.065161 | 3.006551 | 0.0040 |
|  |  |  |  |  |
|  |  |  |  |  |
|  | Effects Specification | |  |  |
|  |  |  | S.D. | Rho |
|  |  |  |  |  |
|  |  |  |  |  |
| Cross-section random | | | 0.905270 | 0.8640 |
| Idiosyncratic random | | | 0.359167 | 0.1360 |
|  |  |  |  |  |
|  |  |  |  |  |
|  | Weighted Statistics | |  |  |
|  |  |  |  |  |
|  |  |  |  |  |
| R-squared | 0.408020 | Mean dependent var | | 0.316054 |
| Adjusted R-squared | 0.353207 | S.D. dependent var | | 0.434934 |
| S.E. of regression | 0.349789 | Sum squared resid | | 6.607031 |
| F-statistic | 7.443867 | Durbin-Watson stat | | 1.970521 |
| Prob(F-statistic) | 0.000023 |  |  |  |
|  |  |  |  |  |
|  |  |  |  |  |
|  | Unweighted Statistics | |  |  |
|  |  |  |  |  |
|  |  |  |  |  |
| R-squared | 0.338039 | Mean dependent var | | 1.809083 |
| Sum squared resid | 38.95833 | Durbin-Watson stat | | 0.334185 |
|  |  |  |  |  |
|  |  |  |  |  |

# Singapore

Chow Test

| Redundant Fixed Effects Tests | | |  |  |
| --- | --- | --- | --- | --- |
| Equation: FEM | |  |  |  |
| Test cross-section fixed effects | | | |  |
|  |  |  |  |  |
|  |  |  |  |  |
| Effects Test | | Statistic | d.f. | Prob. |
|  |  |  |  |  |
|  |  |  |  |  |
| Cross-section F | | 74.276465 | (7,27) | 0.0000 |
| Cross-section Chi-square | | 120.339743 | 7 | 0.0000 |
|  |  |  |  |  |
|  |  |  |  |  |

Hausman Test

| Correlated Random Effects - Hausman Test | | | |  |  |
| --- | --- | --- | --- | --- | --- |
| Equation: REM | |  |  |  |  |
| Test cross-section random effects | | | |  |  |
|  |  |  |  |  |  |
|  |  |  |  |  |  |
| Test Summary | | Chi-Sq. Statistic | Chi-Sq. d.f. | Prob. |  |
|  |  |  |  |  |  |
|  |  |  |  |  |  |
| Cross-section random | | 0.000000 | 5 | 1.0000 |  |
|  |  |  |  |  |  |
|  |  |  |  |  |  |

**Table 9. Singapore Panel Regression Result.**

| Dependent Variable: Y_TQ | | |  |  |
| --- | --- | --- | --- | --- |
| Method: Panel EGLS (Cross-section random effects) | | | | |
| Date: 07/29/22 Time: 11:06 | | |  |  |
| Sample: 2017 2021 | | |  |  |
| Periods included: 5 | | |  |  |
| Cross-sections included: 8 | | |  |  |
| Total panel (balanced) observations: 40 | | | |  |
| Swamy and Arora estimator of component variances | | | | |
|  |  |  |  |  |
|  |  |  |  |  |
| Variable | Coefficient | Std. Error | t-Statistic | Prob. |
|  |  |  |  |  |
|  |  |  |  |  |
| C | 6.759654 | 1.454642 | 4.646954 | 0.0000 |
| X1_TE | 0.659939 | 0.182289 | 3.620300 | 0.0009 |
| X2_SIZESALES | -0.253939 | 0.070001 | -3.627666 | 0.0009 |
| X3_DAR | -0.956616 | 0.516292 | -1.852858 | 0.0726 |
| X4_GROWTH | 0.054649 | 0.279527 | 0.195505 | 0.8462 |
| X10_INF | -0.066247 | 0.037735 | -1.755576 | 0.0882 |
|  |  |  |  |  |
|  |  |  |  |  |
|  | Effects Specification | |  |  |
|  |  |  | S.D. | Rho |
|  |  |  |  |  |
|  |  |  |  |  |
| Cross-section random | | | 0.200979 | 0.5215 |
| Idiosyncratic random | | | 0.192524 | 0.4785 |
|  |  |  |  |  |
|  |  |  |  |  |
|  | Weighted Statistics | |  |  |
|  |  |  |  |  |
|  |  |  |  |  |
| R-squared | 0.180094 | Mean dependent var | | 0.586386 |
| Adjusted R-squared | 0.059520 | S.D. dependent var | | 0.411208 |
| S.E. of regression | 0.398783 | Sum squared resid | | 5.406949 |
| F-statistic | 1.493634 | Durbin-Watson stat | | 0.442487 |
| Prob(F-statistic) | 0.217737 |  |  |  |
|  |  |  |  |  |
|  |  |  |  |  |
|  | Unweighted Statistics | |  |  |
|  |  |  |  |  |
|  |  |  |  |  |
| R-squared | 0.256819 | Mean dependent var | | 1.489100 |
| Sum squared resid | 26.57506 | Durbin-Watson stat | | 0.090028 |
|  |  |  |  |  |
|  |  |  |  |  |

# Pakistan

Chow Test

| Redundant Fixed Effects Tests | | |  |  |
| --- | --- | --- | --- | --- |
| Equation: FEM | |  |  |  |
| Test cross-section fixed effects | | | |  |
|  |  |  |  |  |
|  |  |  |  |  |
| Effects Test | | Statistic | d.f. | Prob. |
|  |  |  |  |  |
|  |  |  |  |  |
| Cross-section F | | 30.010459 | (6,23) | 0.0000 |
| Cross-section Chi-square | | 76.230730 | 6 | 0.0000 |
|  |  |  |  |  |
|  |  |  |  |  |
|  |  |  |  |  |

Hausman Test

| Correlated Random Effects - Hausman Test | | | |  |
| --- | --- | --- | --- | --- |
| Equation: REM | |  |  |  |
| Test cross-section random effects | | | |  |
|  |  |  |  |  |
|  |  |  |  |  |
| Test Summary | | Chi-Sq. Statistic | Chi-Sq. d.f. | Prob. |
|  |  |  |  |  |
|  |  |  |  |  |
| Cross-section random | | 3.876842 | 5 | 0.5673 |
|  |  |  |  |  |
|  |  |  |  |  |

Lagrange Multiplier Test

| Lagrange Multiplier Tests for Random Effects | | | |
| --- | --- | --- | --- |
| Null hypotheses: No effects | | |  |
| Alternative hypotheses: Two-sided (Breusch-Pagan) and one-sided | | | |
| (all others) alternatives | | |  |
|  |  |  |  |
|  |  |  |  |
|  | Test Hypothesis | | |
|  | Cross-section | Time | Both |
|  |  |  |  |
|  |  |  |  |
| Breusch-Pagan | 40.43217 | 2.507964 | 42.94013 |
|  | (0.0000) | (0.1133) | (0.0000) |

**Table 10. Pakistan Panel Regression Result.**

| Dependent Variable: Y_TQ | | |  |  |
| --- | --- | --- | --- | --- |
| Method: Panel EGLS (Cross-section random effects) | | | | |
| Date: 07/30/22 Time: 14:08 | | |  |  |
| Sample: 2017 2021 | | |  |  |
| Periods included: 5 | | |  |  |
| Cross-sections included: 7 | | |  |  |
| Total panel (balanced) observations: 35 | | | |  |
| Swamy and Arora estimator of component variances | | | | |
|  |  |  |  |  |
|  |  |  |  |  |
| Variable | Coefficient | Std. Error | t-Statistic | Prob. |
|  |  |  |  |  |
|  |  |  |  |  |
| C | -32.60425 | 26.95107 | -1.209757 | 0.2361 |
| X1_TE | 1.546922 | 1.205908 | 1.282786 | 0.2097 |
| X2_SIZESALES | 1.865394 | 1.424823 | 1.309212 | 0.2007 |
| X3_DAR | 2.093301 | 2.842987 | 0.736303 | 0.4675 |
| X4_GROWTH | 1.211664 | 1.572798 | 0.770387 | 0.4473 |
| X10_INF | -0.103180 | 0.073921 | -1.395805 | 0.1734 |
|  |  |  |  |  |
|  |  |  |  |  |
|  | Effects Specification | |  |  |
|  |  |  | S.D. | Rho |
|  |  |  |  |  |
|  |  |  |  |  |
| Cross-section random | | | 3.729524 | 0.9182 |
| Idiosyncratic random | | | 1.113321 | 0.0818 |
|  |  |  |  |  |
|  |  |  |  |  |
|  | Weighted Statistics | |  |  |
|  |  |  |  |  |
|  |  |  |  |  |
| R-squared | 0.258962 | Mean dependent var | | 0.579573 |
| Adjusted R-squared | 0.131197 | S.D. dependent var | | 1.191888 |
| S.E. of regression | 1.110954 | Sum squared resid | | 35.79236 |
| F-statistic | 2.026860 | Durbin-Watson stat | | 1.609908 |
| Prob(F-statistic) | 0.104329 |  |  |  |
|  |  |  |  |  |
|  |  |  |  |  |
|  | Unweighted Statistics | |  |  |
|  |  |  |  |  |
|  |  |  |  |  |
| R-squared | -0.024294 | Mean dependent var | | 4.379879 |
| Sum squared resid | 362.9636 | Durbin-Watson stat | | 0.158755 |
|  |  |  |  |  |
|  |  |  |  |  |

# South Africa

Chow Test

| Redundant Fixed Effects Tests | | |  |  |
| --- | --- | --- | --- | --- |
| Equation: FEM | |  |  |  |
| Test cross-section fixed effects | | | |  |
|  |  |  |  |  |
|  |  |  |  |  |
| Effects Test | | Statistic | d.f. | Prob. |
|  |  |  |  |  |
|  |  |  |  |  |
| Cross-section F | | 1.395890 | (7,27) | 0.2475 |
| Cross-section Chi-square | | 12.355158 | 7 | 0.0895 |
|  |  |  |  |  |
|  |  |  |  |  |

Lagrange Multiplier Test

| Lagrange Multiplier Tests for Random Effects | | | |
| --- | --- | --- | --- |
| Null hypotheses: No effects | | |  |
| Alternative hypotheses: Two-sided (Breusch-Pagan) and one-sided | | | |
| (all others) alternatives | | |  |
|  |  |  |  |
|  |  |  |  |
|  | Test Hypothesis | | |
|  | Cross-section | Time | Both |
|  |  |  |  |
|  |  |  |  |
| Breusch-Pagan | 0.049550 | 5.45E-05 | 0.049604 |
|  | (0.8238) | (0.9941) | (0.8238) |
|  |  |  |  |

**Table 11. South Africa Panel Regression Result.**

| Dependent Variable: Y_TQ | | |  |  |
| --- | --- | --- | --- | --- |
| Method: Panel Least Squares | | |  |  |
| Date: 07/30/22 Time: 13:50 | | |  |  |
| Sample: 2017 2021 | | |  |  |
| Periods included: 5 | | |  |  |
| Cross-sections included: 8 | | |  |  |
| Total panel (balanced) observations: 40 | | | |  |
|  |  |  |  |  |
|  |  |  |  |  |
| Variable | Coefficient | Std. Error | t-Statistic | Prob. |
|  |  |  |  |  |
|  |  |  |  |  |
| C | -0.800444 | 2.727229 | -0.293501 | 0.7709 |
| X1_TE | 2.220265 | 0.937911 | 2.367245 | 0.0238 |
| X2_SIZESALES | -0.058621 | 0.113978 | -0.514322 | 0.6104 |
| X3_DAR | 1.265481 | 1.231064 | 1.027958 | 0.3112 |
| X4_GROWTH | -1.254290 | 1.252117 | -1.001736 | 0.3235 |
| X10_INF | 0.373466 | 0.305933 | 1.220741 | 0.2306 |
|  |  |  |  |  |
|  |  |  |  |  |
| R-squared | 0.194566 | Mean dependent var | | 1.482125 |
| Adjusted R-squared | 0.076120 | S.D. dependent var | | 1.216370 |
| S.E. of regression | 1.169159 | Akaike info criterion | | 3.287927 |
| Sum squared resid | 46.47568 | Schwarz criterion | | 3.541259 |
| Log likelihood | -59.75853 | Hannan-Quinn criter. | | 3.379524 |
| F-statistic | 1.642655 | Durbin-Watson stat | | 1.275129 |
| Prob(F-statistic) | 0.175379 |  |  |  |
|  |  |  |  |  |
|  |  |  |  |  |
