# Supplementary material for: The impact of technical efficiency on firms’ value: The case of the halal food and beverage industry in selected countries
Source: PLoS One. 2023 Nov 27;18(11):e0286629. doi: 10.1371/journal.pone.0286629 (PMC10681182; doi:10.1371/journal.pone.0286629)
Supplement: S2 File — (DOCX) [file pone.0286629.s004.docx]

**Appendix 1. Malaysia List of Sample**

| **Firm Code** | | | | **2017** | | **2018** | | **2019** | **2020** | | **2021** | | **Mean** |
| --- | --- | --- | --- | --- | --- | --- | --- | --- | --- | --- | --- | --- | --- |
| Hub Seng Industries Bhd | | | | 0.861 | | 0.860 | | 0.815 | 0.834 | | 0.753 | | 0.824 |
| Fraser & Neave Holdings Bhd | | | | 0.604 | | 0.722 | | 0.832 | 0.858 | | 0.816 | | 0.767 |
| Nestle Malaysia Bhd | | | | 0.831 | | 0.806 | | 0.861 | 0.668 | | 0.661 | | 0.765 |
| Ajinomoto Malaysia Bhd | | | | 0.851 | | 0.812 | | 0.764 | 0.472 | | 0.895 | | 0.759 |
| Dutch Lady Milk Industries Bhd | | | | 0.792 | | 0.787 | | 0.734 | 0.528 | | 0.943 | | 0.757 |
| Cocoaland Holdings Bhd | | | | 0.802 | | 0.677 | | 0.759 | 0.565 | | 0.296 | | 0.620 |
| ABLE Global Bhd | | | | 0.460 | | 0.506 | | 0.709 | 0.628 | | 0.741 | | 0.609 |
| Three-A Resources Bhd | | | | 0.900 | | 0.554 | | 0.710 | 0.687 | | 0.185 | | 0.607 |
| Kawan Food Bhd | | | | 0.796 | | 0.687 | | 0.324 | 0.590 | | 0.636 | | 0.607 |
| CCK Consolidated Holdings Bhd | | | | 0.566 | | 0.517 | | 0.595 | 0.619 | | 0.389 | | 0.537 |
| Spritzer Bhd | | | | 0.521 | | 0.495 | | 0.678 | 0.629 | | 0.151 | | 0.495 |
| Apollo Food Holdings Bhd | | | | 0.424 | | 0.396 | | 0.571 | 0.474 | | 0.536 | | 0.480 |
| Power Root Bhd | | | | 0.159 | | 0.509 | | 0.617 | 0.488 | | 0.500 | | 0.454 |
| Oriental Food Industries Holdings Bhd | | | | 0.233 | | 0.414 | | 0.352 | 0.517 | | 0.542 | | 0.412 |
| C.I. Holdings Bhd | | | | 0.246 | | 0.209 | | 0.228 | 0.206 | | 0.461 | | 0.270 |
| Guan Chong Bhd | | | | 0.156 | | 0.318 | | 0.420 | 0.265 | | 0.064 | | 0.245 |
| Malayan Flour Mills Bhd | | | | 0.187 | | 0.128 | | 0.134 | 0.085 | | 0.153 | | 0.137 |
| Mean | | | | 0.552 | | 0.553 | | 0.594 | 0.536 | | 0.513 | | 0.550 |
|  |  |  |  | |  | |  | | |  | |  |  |

5%=1.989686

**Appendix 2. Indonesia List of Sample**

| **Firm Code** | **2017** | **2018** | **2019** | **2020** | **2021** | **Mean** |
| --- | --- | --- | --- | --- | --- | --- |
| Akasha Wira International Tbk. | 0.714 | 0.709 | 0.837 | 0.799 | 1.000 | 0.812 |
| Mayora Indah Tbk. | 0.996 | 0.927 | 0.919 | 0.721 | 0.451 | 0.803 |
| Siantar Top Tbk. | 0.961 | 0.744 | 0.948 | 0.848 | 0.474 | 0.795 |
| Indofood CBP Sukses Makmur Tbk. | 0.660 | 0.794 | 0.763 | 0.903 | 0.716 | 0.767 |
| Tunas Baru Lampung Tbk. | 0.970 | 0.810 | 0.662 | 0.627 | 0.606 | 0.735 |
| Sekar Laut Tbk. | 0.558 | 0.576 | 0.784 | 0.701 | 0.688 | 0.661 |
| Ultra Jaya Milk Industry & Tea Tbk. | 0.552 | 0.364 | 0.429 | 0.823 | 0.918 | 0.617 |
| Indofood Sukses Makmur Tbk. | 0.514 | 0.523 | 0.597 | 0.656 | 0.639 | 0.586 |
| Wilmar Cahaya Indonesia Tbk. | 0.656 | 0.349 | 0.754 | 0.572 | 0.561 | 0.578 |
| BUDI | 0.513 | 0.437 | 0.595 | 0.485 | 0.503 | 0.507 |
| Nippon Indosari Corpindo Tbk. | 0.268 | 0.210 | 0.386 | 0.254 | 0.759 | 0.375 |
| Sekar Bumi Tbk. | 0.172 | 0.154 | 0.175 | 0.263 | 0.266 | 0.206 |
| Mean | 0.628 | 0.550 | 0.654 | 0.638 | 0.632 | 0.620 |

5% = 2.003240719

**Appendix 3. Singapore List of Sample**

| **Firm Code** | **2017** | **2018** | **2019** | **2020** | **2021** | **Mean** |
| --- | --- | --- | --- | --- | --- | --- |
| Shen Shiong Group Ltd | 0.959 | 0.895 | 0.751 | 0.996 | 0.921 | 0.904 |
| Japfa Ltd | 0.484 | 0.857 | 0.756 | 0.686 | 0.406 | 0.638 |
| Bumitama Agri Ltd | 0.654 | 0.568 | 0.313 | 0.568 | 1.000 | 0.621 |
| First Resources Ltd | 0.660 | 0.658 | 0.492 | 0.555 | 0.719 | 0.617 |
| JB Foods Ltd | 0.574 | 0.873 | 0.560 | 0.406 | 0.300 | 0.542 |
| Old Chang Kee Ltd | 0.314 | 0.415 | 0.206 | 0.457 | 0.396 | 0.358 |
| QAF Ltd | 0.196 | 0.034 | 0.180 | 0.625 | 0.628 | 0.333 |
| Fraser & Neave Ltd | 0.096 | 0.164 | 0.216 | 0.196 | 0.197 | 0.174 |
| Mean | 0.492 | 0.558 | 0.434 | 0.561 | 0.571 | 0.523 |

5% = 2.028094001

**Appendix 4. Pakistan List of Sample**

| **Firm Code** | | | **2017** | | **2018** | | **2019** | **2020** | | **2021** | | **Mean** |
| --- | --- | --- | --- | --- | --- | --- | --- | --- | --- | --- | --- | --- |
| Rafhan Maize Products Co Ltd | | | 0.808 | | 0.781 | | 0.730 | 0.633 | | 0.651 | | 0.721 |
| Unilever Pakistan Foods Ltd | | | 0.342 | | 0.752 | | 0.815 | 0.853 | | 0.713 | | 0.695 |
| NESTLE Pakistan Ltd | | | 0.840 | | 0.622 | | 0.465 | 0.705 | | 0.703 | | 0.667 |
| Ismail Industries Ltd | | | 0.774 | | 0.774 | | 0.546 | 0.466 | | 0.541 | | 0.620 |
| Murree Brewery Company Ltd | | | 0.661 | | 0.653 | | 0.532 | 0.405 | | 0.570 | | 0.564 |
| National Foods Limited | | | 0.411 | | 0.483 | | 0.445 | 0.553 | | 0.391 | | 0.457 |
| Friesland Campina Engro Pakistan Ltd | | | 0.643 | | 0.283 | | 0.131 | 0.520 | | 0.603 | | 0.436 |
| Mean | | | 0.640 | | 0.621 | | 0.523 | 0.591 | | 0.596 | | 0.594 |
|  |  |  |  |  | |  | | |  | |  |  |

5% = 2.039513

**Appendix 5. South Africa List of Sample**

| **Firm Code** | **2017** | **2018** | **2019** | **2020** | **2021** | **Mean** |
| --- | --- | --- | --- | --- | --- | --- |
| AVI Ltd | 0.815 | 0.738 | 0.683 | 0.680 | 0.774 | 0.738 |
| Sea Harvest Group Ltd | 0.904 | 0.581 | 0.834 | 0.868 | 0.298 | 0.697 |
| AH-Vest Ltd | 0.351 | 0.338 | 0.681 | 0.885 | 0.769 | 0.605 |
| Oceana Group Ltd | 0.446 | 0.486 | 0.612 | 0.668 | 0.744 | 0.591 |
| Premier Fishing and Brands Ltd | 0.382 | 0.770 | 0.895 | 0.339 | 0.422 | 0.561 |
| Crookers Brothers Ltd | 0.044 | 0.597 | 0.341 | 0.666 | 0.739 | 0.477 |
| Tiger Brands Ltd | 0.765 | 0.509 | 0.445 | 0.279 | 0.358 | 0.471 |
| RFG Holdings Ltd | 0.478 | 0.317 | 0.407 | 0.410 | 0.324 | 0.387 |
| Mean | 0.523 | 0.542 | 0.612 | 0.599 | 0.554 | 0.566 |

5% = 2.028094001
