# Supplementary material for: The impact of technical efficiency on firms’ value: The case of the halal food and beverage industry in selected countries
Source: PLoS One. 2023 Nov 27;18(11):e0286629. doi: 10.1371/journal.pone.0286629 (PMC10681182; doi:10.1371/journal.pone.0286629)
Supplement: Attachment — Submitted filename: Comments Plos One Journal.pdf [file pone.0286629.s007.pdf]

Title: I suggest that the title of this manuscript should be "The impact of technical efficiency on the Firms' value: the case of the Halal food and beverage industry in selected countries".

Abstract: This section has been descriptively written. It is suggested to include the findings of the study here. In addition, policy implications should be included concisely at the end of this section.

Introduction: Data used in this section should be updated to most recent years. Organization of remaining of this study should be outlined at the end of this section.

Literature Review: This section should be divided into three parts. The first part must be based on the theoretical background. Secondly, the discussion should be developed on the estimation of technical efficiency. DEA and SFA approaches are used to estimate technical efficiency. This study employed the SFA approach to calculate the technical efficiency of halal food and beverages. Please justify the SFA approach compared to the DEA approach. Consult the latest published articles for your guidance and improve this section (https://doi.org/10.3390/en14133923; https://doi.org/10.1007/s10668-020-01023-2; https://doi.org/10.3389/fenrg.2022.943771; https://doi.org/10.1007/s10668-022-02194-w). Third, include empirical literature that explores the impact of efficiency on firm value. Finally, find the research gap that this study fills.

Data and Statistical Method: (1). The title of this section should be "Methodology". (2). It is recommended to include a table regarding "description of the data series" used in this study. (3). It is recommended to justify the significance of each variable included in the model. (4). Technical efficiency is estimated from the production function including capital, labor and other factors of the firm. This study is missing labor of the firm.

Results and Discussion: first, it is recommended to include descriptive statistics of the variables for each country used in the study in tabular form. This study is based on panel data with five cross sections and time period 2017-2021. Use the panel data SFA model to calculate the technical efficiency of each country with different time periods. It is recommended to use the above-mentioned articles for your guidance and to improve this section. Second, report findings of the SFA panel data model along with diagnostic statistics. Third, report each country's technical efficiency along with their ranking. Finally, to explore the impact of technical efficiency and other control variables on firm performance, use panel data models such as fixed effect, random effect, ARDL panel, or fully modified dynamic panel model, whichever best fits your data series. It is recommended to merge the discussion section with the "Results and Discussion" section.

Conclusion: This section should be titled "Conclusions and policy Implications". Include policy implications based on study results. It is recommended to include "Future Research Directions" at the end of this section.
