# Supplementary material for: The impact of technical efficiency on firms’ value: The case of the halal food and beverage industry in selected countries
Source: PLoS One. 2023 Nov 27;18(11):e0286629. doi: 10.1371/journal.pone.0286629 (PMC10681182; doi:10.1371/journal.pone.0286629)
Supplement: Attachment — Submitted filename: Response to reviewers.pdf [file pone.0286629.s008.pdf]

**Date:**	Feb 13 2023 09:17AM
**To:**	"Sylva Alif Rusmita" sylvalifr@feb.unair.ac.id
**cc:**	nemer.badwan@mail.ru
**From:**	"PLOS ONE" plosone@plos.org
**Subject:**	PLOS ONE Decision: Revision required [PONE-D-22-31308R1]
**Attachment(s):**	Comments Plos One Journal.pdf

PONE-D-22-31308R1
EFFICIENCY LEVEL OF HALAL FOOD AND BEVERAGE COMPANIES AND MARKET VALUE IN SELECTED COUNTRIES
PLOS ONE

Dear Dr. Sylva Alif Rusmita,

Thank you for submitting your manuscript to PLOS ONE. After careful consideration, we feel that it has merit but does not fully meet PLOS ONE's publication criteria as it currently stands. Therefore, we invite you to submit a revised version of the manuscript that addresses the points raised during the review process.

==============================

Please submit your revised manuscript by 28 February, 2023. If you will need more time than this to complete your revisions, please reply to this message or contact the journal office at plosone@plos.org. When you're ready to submit your revision, log on to https://www.editorialmanager.com/pone/ and select the 'Submissions Needing Revision' folder to locate your manuscript file.

Please include the following items when submitting your revised manuscript:

- A rebuttal letter that responds to each point raised by the academic editor and reviewer(s). You should upload this letter as a separate file labeled 'Response to Reviewers'.
- A marked-up copy of your manuscript that highlights changes made to the original version. You should upload this as a separate file labeled 'Revised Manuscript with Track Changes'.
- An unmarked version of your revised paper without tracked changes. You should upload this as a separate file labeled 'Manuscript'.

If you would like to make changes to your financial disclosure, please include your updated statement in your cover letter. Guidelines for resubmitting your figure files are available below the reviewer comments at the end of this letter.

If applicable, we recommend that you deposit your laboratory protocols in protocols.io to enhance the reproducibility of your results. Protocols.io assigns your protocol its own identifier (DOI) so that it can be cited independently in the future. For instructions see: https://journals.plos.org/plosone/s/submission-guidelines#loc-laboratory-protocols. Additionally, PLOS ONE offers an option for publishing peer-reviewed Lab Protocol articles, which describe protocols hosted on protocols.io. Read more information on sharing protocols at https://plos.org/protocols?utm_medium=editorial-email&utm_source=authorletters&utm_campaign=protocols.

We look forward to receiving your revised manuscript.

Kind Regards,

Asst. Prof. Dr. Nemer Badwan

PhD in Economics and Finance

Assistant Professor of Economics and Finance
Academic Editor
PLOS ONE

[Note: HTML markup is below. Please do not edit.]

Reviewers' comments:

Reviewer's Responses to Questions

Comments to the Author

1. If the authors have adequately addressed your comments raised in a previous round of review and you feel that this manuscript is now acceptable for publication, you may indicate that here to bypass the "Comments to the Author" section, enter your conflict of interest statement in the "Confidential to Editor" section, and submit your "Accept" recommendation.

Reviewer #3: All comments have been addressed

Reviewer #4: (No Response)

2. Is the manuscript technically sound, and do the data support the conclusions?

The manuscript must describe a technically sound piece of scientific research with data that supports the conclusions. Experiments must have been conducted rigorously, with appropriate controls, replication, and sample sizes. The conclusions must be drawn appropriately based on the data presented.

Reviewer #3: Yes

Reviewer #4: Partly

---

3. Has the statistical analysis been performed appropriately and rigorously?

Reviewer #3: Yes

Reviewer #4: No

---

4. Have the authors made all data underlying the findings in their manuscript fully available?

The PLOS Data policy requires authors to make all data underlying the findings described in their manuscript fully available without restriction, with rare exception (please refer to the Data Availability Statement in the manuscript PDF file). The data should be provided as part of the manuscript or its supporting information, or deposited to a public repository. For example, in addition to summary statistics, the data points behind means, medians and variance measures should be available. If there are restrictions on publicly sharing data—e.g. participant privacy or use of data from a third party—those must be specified.

Reviewer #3: Yes

Reviewer #4: Yes

---

5. Is the manuscript presented in an intelligible fashion and written in standard English?

PLOS ONE does not copyedit accepted manuscripts, so the language in submitted articles must be clear, correct, and unambiguous. Any typographical or grammatical errors should be corrected at revision, so please note any specific errors here.

Reviewer #3: Yes

Reviewer #4: No

---

6. Review Comments to the Author

Please use the space provided to explain your answers to the questions above. You may also include additional comments for the author, including concerns about dual publication, research ethics, or publication ethics. (Please upload your review as an attachment if it exceeds 20,000 characters)

Reviewer #3: Dear Authors,
thank you for addressing the previous elements.
What I can suggest to fix and create a better conclusion and implications section.
Particularly, what I expect is a holistic section with some sub-paragraphs as Theoretical Implications and Practical Implications. Then, more practical implications should be added.

All the best
The reviewer

Thanks for the suggestion, we explain this part at 3th paragraph of conclusion and policy implication section. For theoretical implication, this research strongly support the signaling theory and emphasize that investor in halal FnB consider as rational investor. Along with that, we also explain the practical implication for several parties such as firm which can be known their operating managerial, benchmark, and remain them to control their TE score, because it is impacting to firm performance in capital market. Furthermore, the implication for policy maker to make the rules like giving intensive to the industry to minimizing the inefficiency that happen in firm, consider the strongest of halal FnB compare to other countries so they can know the comparative advantages. At the same time this research also beneficial for investor to use TE score as consideration to make decision making because it can impact their wealth as well.

Reviewer #4: Title: I suggest that the title of this manuscript should be "The impact of technical efficiency on the Firms' value: the case of the Halal food and beverage industry in selected countries".

We re-name the title following your advice.

Abstract: This section has been descriptively written. It is suggested to include the findings of the study here. In addition, policy implications should be included concisely at the end of this section.
Thanks for the suggestion. We edit the result by mention each country efficiency score and explain the inefficiency score as well which indicate this industry need further improvement especially form companies managerial side. For policy implication we emphasize this research can beneficially help government to support this sector such as giving subsidy to boost their performance and to identify the strength of industry by comparing TE score of each country.

Introduction: Data used in this section should be updated to most recent years. Organization of remaining of this study should be outlined at the end of this section.

-We are so sorry for not updated the data, this is due to the availability of data in 2022 still have not been publish completely until today by several companies. If we try to add the research timeline until 2022, it will decrease number of research sample. Hope you can consider this part.
-We add the organization of the article at the end of introduction part.

Literature Review: This section should be divided into three parts. The first part must be based on the theoretical background. Secondly, the discussion should be developed on the estimation of technical efficiency. DEA and SFA approaches are used to estimate technical efficiency. This study employed the SFA approach to calculate the technical efficiency of halal food and beverages. Please justify the SFA approach compared to the DEA approach. Consult the latest published articles for your guidance and improve this section (https://doi.org/10.3390/en14133923; https://doi.org/10.1007/s10668-020-01023-2; https://doi.org/10.3389/fenrg.2022.943771; https://doi.org/10.1007/s10668-022-02194-w). Third, include empirical literature that explores the impact of efficiency on firm value. Finally, find the research gap that this study fills.
We rewrite this sections following your comment. We separated the literature review into 3 part and close it with hypothesis. The theoretical background that we used is signaling theory, then for technical efficiency section, we emphasize the differences of DEA and SFA as well as the reason for choosing SFA approach for two steps estimation model. We also try to re-write the empirical review which support this research and find the research gaps which are lies in several factors.

Data and Statistical Method: (1). the title of this section should be "Methodology". (2). It is recommended to include a table regarding "description of the data series" used in this study. (3). It is recommended to justify the significance of each variable included in the model. (4). Technical efficiency is estimated from the production function including capital, labor and other factors of the firm. This study is missing labor of the firm.
Thanks for the recommendation.
- We re-new the section by "Methodology".
- We also explain the data series in Table 1 and describe every variable that has been used after the equation as well as connecting the reason for choosing the variables based on previous study.
- We add the significant of each variable (at 5%) in every sub section of variables.
- For labor variable, we are apologize for not including in our model due to several reasons: (1) The labor cost already include in liability variable, if we input again it will cause double counting; (2) we already try to separate between liability and labor cost, but the panel model shows the insignificant of TE partially and simultaneously as well as low R-square for each model.

Results and Discussion: first, it is recommended to include descriptive statistics of the variables for each country used in the study in tabular form. This study is based on panel data with five cross sections and time period 2017-2021. Usethe panel data SFA model to calculate the technical efficiency of each country with different time periods. It is recommended to use the above-mentioned articles for your guidance and to improve this section. Second, report findings of the SFA panel data model along with diagnostic statistics. Third, report each country's technical efficiency along with their ranking. Finally, to explore the impact of technical efficiency and other control variables on firm performance, use panel data models such as fixed effect, random effect, ARDL panel, or fully modified dynamic panel model, whichever best fits your data series. It is recommended to merge the discussion section with the "Results and Discussion" section.

Thanks for the suggestions

-We add the statistic descriptive table for SFA and Panel

-We cite the papers that you suggest in literature review and methodology parts.

-We explain the diagnostic statistic of each country to explain the reason of panel best model that we used.

-We rank the country from the highest to the lowest of TE score, which can be seen at table 8

- We edit the result and discussion section. At result, we explain the number, significances, and the impact of significant variable. Whereas, in discussion we try to collaborate the result with actual condition, grand theory that we used, as well as the previous study that support our finding.

Conclusion: This section should be titled "Conclusions and policy Implications". Include policy implications based on study results. It is recommended to include "Future Research Directions" at the end of this section.
-we rename this part as Conclusion and policy implications. At first paragraph we briefly explain the aim of study and its result, than following by explain the panel data approach result and suggestion. The third paragraph we emphasize the research implication for several parties (Firm, investor, and policy makers. In the end we provide the research limitation which also can be used as further direction for researcher.

---

1. PLOS authors have the option to publish the peer review history of their article (what does this mean?). If published, this will include your full peer review and any attached files.

   If you choose "no", your identity will remain anonymous but your review may still be made public.

   Do you want your identity to be public for this peer review? For information about this choice, including consent withdrawal, please see our Privacy Policy.

   Reviewer #3: No

   Reviewer #4: **Yes:** Prof. Dr. Dilawar Khan

[NOTE: If reviewer comments were submitted as an attachment file, they will be attached to this email and accessible via the submission site. Please log into your account, locate the manuscript record, and check for the action link "View Attachments". If this link does not appear, there are no attachment files.]

While revising your submission, please upload your figure files to the Preflight Analysis and Conversion Engine (PACE) digital diagnostic tool, https://pacev2.apexcovantage.com/. PACE helps ensure that figures meet PLOS requirements. To use PACE, you must first register as a user. Registration is free. Then, login and navigate to the UPLOAD tab, where you will find detailed instructions on how to use the tool. If you encounter any issues or have any questions when using PACE, please email PLOS at figures@plos.org. Please note that Supporting Information files do not need this step.

In compliance with data protection regulations, you may request that we remove your personal registration details at any time. (Remove my information/details). Please contact the publication office if you have any questions.
