# Supplementary material for: The impact of technical efficiency on firms’ value: The case of the halal food and beverage industry in selected countries
Source: PLoS One. 2023 Nov 27;18(11):e0286629. doi: 10.1371/journal.pone.0286629 (PMC10681182; doi:10.1371/journal.pone.0286629)
Supplement: Attachment — Submitted filename: Review report PONE.docx [file pone.0286629.s005.docx]

Review report PONE-D-22-31308

Efficiency level of food and beverage companies and market value in selected countries

**Topic**

The author should highlight Islamic (Halal) food and beverage companies in the topic.

**Introduction**

Row 72, halal food and beverage companies in the 11 best countries. Best countries in terms of?

**Literature Review**

It is inappropriate to include current assets, fixed assets, liabilities, equity, operating income, firm value as the sub-sections under the Literature Review section. Since they are the variables of this study, it is more appropriate to discuss them under the Methodology section.

**Previous research**

The authors should include the literature on the food and beverage industry.

**Hypothesis**

H1 is not a hypothesis. It is just an interpretation of the efficiency score. Please remove it.

Usually, each hypothesis is developed from a theory. H2 is developed from which theory?

**Method**

Row 241, the paper mentions the top 11 halal food countries. Row 244, it mentions the top 5 countries. So it is 11 or 5? Inconsistent data presentation. These 51 firms are listed companies? Please provide a list of company names as an appendix. The authors should mention which software they use to compute the results for step 1 and step 2.

**Result**

Since the authors report the result for each country, there should be a list of sample size for each country under the method section.

Row 330, showed a relatively stable result of between 0.513 and 0.55. Is this information obtained from Table 1? Please elaborate it.

The authors show so many results in different tables, but the text does not elaborate on the content well. Please improve it.

Table 3, 6, 7, 9, 10, 12, 13 and 15. The authors should not write the coefficient and independent symbol together. That is not the appropriate way to present the panel regression model.

Row 503, Table 18, it is inappropriate to report the p-value under the result column. Which text is presenting Table 18?

**Conclusion**

Since the authors do not present the result and discussion sections well, it is difficult to comment on the conclusion section. Therefore, the authors need to put more effort to improve the result and discussion sections.
