# Supplementary material for: The impact of technical efficiency on firms’ value: The case of the halal food and beverage industry in selected countries
Source: PLoS One. 2023 Nov 27;18(11):e0286629. doi: 10.1371/journal.pone.0286629 (PMC10681182; doi:10.1371/journal.pone.0286629)
Supplement: Attachment — Submitted filename: Response to Reviewers.docx [file pone.0286629.s006.docx]

**Date:**	Dec 12 2022 05:32PM

**To:**	"Sylva Alif Rusmita" [sylvalifr@feb.unair.ac.id](mailto:sylvalifr@feb.unair.ac.id)

**cc:**	[nemer.badwan@mail.ru](mailto:nemer.badwan@mail.ru)

**From:**	"PLOS ONE" [plosone@plos.org](mailto:plosone@plos.org)

**Subject:**	PLOS ONE Decision: Revision required [PONE-D-22-31308]

**Attachment(s):** [Review report PONE.docx](https://www.editorialmanager.com/pone/download.aspx?guid=%7B98974e45-7da3-4b92-b655-c0757b4e08ee%7D&scheme=13&id=485629)

PONE-D-22-31308

EFFICIENCY LEVEL OF FOOD AND BEVERAGE COMPANIES AND MARKET VALUE IN SELECTED COUNTRIES PLOS ONE

Dear Dr. Rusmita,

Thank you for submitting your manuscript to PLOS ONE. After careful consideration, we feel that it has merit but does not fully meet PLOS ONE’s publication criteria as it currently stands. Therefore, we invite you to submit a revised version of the manuscript that addresses the points raised during the review process.

Please submit your revised manuscript by December 26, 2022. If you will need more time than this to complete your revisions, please reply to this message or contact the journal office at [plosone@plos.org](mailto:plosone@plos.org). When you're ready to submit your revision, log on to https[://w](http://www.editorialmanager.com/pone/)ww[.ed](http://www.editorialmanager.com/pone/)i[torialmanager.com/pone/](http://www.editorialmanager.com/pone/) and select the 'Submissions Needing Revision' folder to locate your manuscript file.

Please include the following items when submitting your revised manuscript:

A rebuttal letter that responds to each point raised by the academic editor and reviewer(s). You should upload this letter as a separate file labeled 'Response to Reviewers'.

A marked-up copy of your manuscript that highlights changes made to the original version. You should upload this as a separate file labeled 'Revised Manuscript with Track Changes'.

An unmarked version of your revised paper without tracked changes. You should upload this as a separate file labeled 'Manuscript'.

If you would like to make changes to your financial disclosure, please include your updated statement in your cover letter. Guidelines for resubmitting your figure files are available below the reviewer comments at the end of this letter.

If applicable, we recommend that you deposit your laboratory protocols in protocols.io to enhance the reproducibility of your results. Protocols.io assigns your protocol its own identifier (DOI) so that it can be cited independently in the future. For instructions see: <https://journals.plos.org/plosone/s/submission-guidelines#loc-laboratory-protocols>.

Additionally, PLOS ONE offers an option for publishing peer-reviewed Lab Protocol articles, which describe protocols [hosted on protocols.io. Read more information on sharing protocols at https://plos.org/protocols? utm_medium=editorial-email&utm_source=authorletters&utm_campaign=protocols.](https://plos.org/protocols?utm_medium=editorial-email&utm_source=authorletters&utm_campaign=protocols)

We look forward to receiving your revised manuscript. Kind regards,

Asst. Prof. Dr. Nemer Badwan

PhD in Economics and Finance

Assistant Professor of Economics and Finance Academic Editor

PLOS ONE

Journal Requirements:

When submitting your revision, we need you to address these additional requirements.

1. Please ensure that your manuscript meets PLOS ONE's style requirements, including those for file naming. The PLOS ONE style templates can be found at https://journals.plos.org/plosone/s/file?id=wjVg/PLOSOne_formatting_sample_main_body.pdf and https://journals.plos.org/plosone/s/file?id=ba62/PLOSOne_formatting_sample_title_authors_affiliations.pdf
2. PLOS ONE does not copy edit accepted manuscripts (https://journals.plos.org/plosone/s/criteria-for-publication#loc- 5). To that effect, please ensure that your submission is free of typos and grammatical errors.
3. Please ensure that you include a title page within your main document. We do appreciate that you have a title page document uploaded as a separate file, however, as per our author guidelines [(h](http://journals.plos.org/plosone/s/submission-guidelines#loc-title-page))t[tp://journals.plos.org/plosone/s/submission-guidelines#loc-title-page)](http://journals.plos.org/plosone/s/submission-guidelines#loc-title-page)) we do require this to be part of the manuscript file itself and not uploaded separately.

Could you therefore please include the title page into the beginning of your manuscript file itself, listing all authors and affiliations.

4. We note you have included a table to which you do not refer in the text of your manuscript. Please ensure that you refer to Tables 2, 3, 5, 6, 7, 8, 9, 11, 12, 14, 15, 16, 17, 18, and S1 in your text; if accepted, production will need this

reference to link the reader to the Table.

5. Please include captions for your Supporting Information files at the end of your manuscript, and update any in-text citations to match accordingly. Please see our Supporting Information guidelines for more information: [http://journals.plos.org/plosone/s/supporting-information.](http://journals.plos.org/plosone/s/supporting-information)

[Note: HTML markup is below. Please do not edit.] Reviewers' comments:

Reviewer's Responses to Questions

**Comments to the Author**

1. Is the manuscript technically sound, and do the data support the conclusions?

The manuscript must describe a technically sound piece of scientific research with data that supports the conclusions. Experiments must have been conducted rigorously, with appropriate controls, replication, and sample sizes. The conclusions must be drawn appropriately based on the data presented.

Reviewer #1: No

Reviewer #2: Yes

2. Has the statistical analysis been performed appropriately and rigorously? Reviewer #1: No

Reviewer #2: Yes

3. Have the authors made all data underlying the findings in their manuscript fully available?

The [PLOS Data policy](http://www.plosone.org/static/policies.action#sharing) requires authors to make all data underlying the findings described in their manuscript fully available without restriction, with rare exception (please refer to the Data Availability Statement in the manuscript PDF file). The data should be provided as part of the manuscript or its supporting information, or deposited to a public repository. For example, in addition to summary statistics, the data points behind means, medians and variance measures should be available. If there are restrictions on publicly sharing data—e.g. participant privacy or use of data from a third party—those must be specified.

Reviewer #1: Yes

Reviewer #2: Yes

4. Is the manuscript presented in an intelligible fashion and written in standard English?

PLOS ONE does not copyedit accepted manuscripts, so the language in submitted articles must be clear, correct, and unambiguous. Any typographical or grammatical errors should be corrected at revision, so please note any specific errors here.

Reviewer #1: Yes

Reviewer #2: No

5. Review Comments to the Author

Please use the space provided to explain your answers to the questions above. You may also include additional comments for the author, including concerns about dual publication, research ethics, or publication ethics. (Please upload your review as an attachment if it exceeds 20,000 characters)

Reviewer #1: Review report PONE-D-22-31308

Efficiency level of food and beverage companies and market value in selected countries

Topic

The author should highlight Islamic (Halal) food and beverage companies in the topic.

We have revised with highlight the Halal term in title and empirical review (line 196-238).

Introduction

Row 72, halal food and beverage companies in the 11 best countries. Best countries in terms of?

We have revised by adding information on the 5 largest halal food producing countries based on the 2020 Global Islamic Economic Report (line 79-81). We have revised from 11 countries to 5 countries according to the research methods chapter.

Literature Review

It is inappropriate to include current assets, fixed assets, liabilities, equity, operating income, firm value as the sub- sections under the Literature Review section. Since they are the variables of this study, it is more appropriate to discuss them under the Methodology section.

We have revised it by adding this section to the research method section (line 342-375) (line 424-440)

Previous research

The authors should include the literature on the food and beverage industry.

We have added a review of 6 articles, namely Henderson (2016) and Secinaro & Calandra (2020), Putri et al (2020), Bhaskaran (2021), Mehraliana (2012), and Cummins (2009) We have written about it at the beginning of the empirical review chapter (line 175-238)

Hypothesis

H1 is not a hypothesis. It is just an interpretation of the efficiency score. Please remove it. Usually, each hypothesis is developed from a theory. H2 is developed from which theory?

We have revised according to the comments. We rewrite the hypothesis part and use the H2, which based on signaling theory hypothesis. We think this hypothesis is relevant because of our paper looking for what the effect of having good efficiency toward firm value (line 276-286)

Method

Row 241, the paper mentions the top 11 halal food countries. Row 244, it mentions the top 5 countries. So it is 11 or 5? Inconsistent data presentation. These 51 firms are listed companies? Please provide a list of company names as an appendix. The authors should mention which software they use to compute the results for step 1 and step 2.

We use the top 5 halal food and beverage companies which take from the global islamic economy report 2020. Then, we do purposive sampling by ignore the country which do not have sharia index from 2017 to 2021 (294-301). For the final sample, we got 5 countries and all of the companies are listed companies. For company name and stock code, we put on the appendix table. We used Frontier 4.1 to calculate the technical efficiency score and Eviews 9 for panel data regression. (We edit following your suggestion in line 310-314)

Result

Since the authors report the result for each country, there should be a list of sample size for each country under the method section.

We adding the table related to list of countries in line 304. List of sample for each countries also explain at panel regression result (table 7-11)

Row 330, showed a relatively stable result of between 0.513 and 0.55. Is this information obtained from Table 1? Please elaborate it.

We rewrite this section for each country at discussion section, technical efficiency part (line 644-724). All the data in this section come from table 12 (The data from table 12 we got from the appendix tables 1-5).

The authors show so many results in different tables, but the text does not elaborate on the content well. Please improve it.

We try to fix the result and discussion section. Each sections we separate into 2 points, first, explain about technical efficiency and second, about panel data related (the effect of efficiency toward firm value). For result section, we explain the data and supporting result from previous study. And for discussion, we analyze the result based on fact and theory which support our finding.

Table 3, 6, 7, 9, 10, 12, 13 and 15. The authors should not write the coefficient and independent symbol together. That is not the appropriate way to present the panel regression model.

Thanks for suggestion. We follow your instruction, please take a look at table 2-11

Row 503, Table 18, it is inappropriate to report the p-value under the result column. Which text is presenting Table 18?

We edit this part, and explain the research column by “significant” or “insignificant”. Please take a look at the new table (table 13, line 739).

Conclusion

Since the authors do not present the result and discussion sections well, it is difficult to comment on the conclusion section. Therefore, the authors need to put more effort to improve the result and discussion sections.

We summarized the result and discussion at conclusion and explain about the implication for companies, investors, government, and future research at implication.

Reviewer #2: The authors try to examine the effect of Technical Efficiency on firm value for the top halal food and beverage firms in 5 countries. They show that improvements in technical efficiency scores positively impact firm values. This study seems important as it studies an underserved topic of halal food and beverages that have not been studied in detail, yet.

Here are my suggestions to the authors.

1. The study needs some reformatting in the structure. There are numerous guides on how to format an academic paper. I suggest the authors read Bellemare (2020) and reformat the paper as much as possible.

We have revised this comment, that is, we follow the chapter numbering and chapter naming according to the Bellemare reference, for example, we changed the grand theory chapter to the theoretical framework chapter, changed the previous study chapter title to empirical review, changed the method chapter title to data and statistical method and so on.

2. The study needs a revamp of the English language formatting. The authors can benefit from sending it to a professional editor to reformat the sentences and paragraphs. While the content of the study is important, it seems like it is written in a very simple language. While there are numerous examples, I will just focus on three simple ones. In the first paragraph of the introduction the authors write “The market share of Muslims in the world as of 2019 was 1.9 billion,”. I believe they meant the number of Muslims, not market share. Then in the second page the authors write “The paper seeks to fill the gap in some of these studies”. I believe the authors mean “fill the gap of the literature”. In page the authors write “The results of this study can be helpful for all parties.” Please explain what these parties are in this sentence.

We did the proofread and re-edit unnecessary sentences. Explanations regarding several parties have been written in the following sentences. The Parties are halal food companies, investors, government, and researcher (line 88-103).

3. Please add a short paragraph at the end of the introduction telling the readers what to expect in the coming section, such as “In section two, we review the literature, while we discuss the methodology in the following section….”

We have added a description of the scheme for the preparation of the sections of this research at the end of the introduction section. (line 117-125)

4. Please merge sections Literature Review and Previous Research.

We apologize for not being able to combine the two because we followed the advice from Reviewer to name and give chapter numbers based on Bellemare's paper (2020). We thank you for your input and comments.

5. The authors need to update the literature review to include the following studies. Henderson (2016) discusses the impact that certification of halal food has on tourism, while Secinaro and Calandra (2020) conducts a detailed review of literature on halal food, which will help the authors in revamping their literature section. In addition, Balli et al (forthcoming) quantifies the extreme connectedness between agricultural commodity prices with food and beverage sector stock market returns.

We have added a review of 2 articles, namely Henderson (2016) and Secinaro & Calandra (2020). We have written about it in line 210-229 of the empirical review chapter. We apologize that we could not find the Bali et al article anywhere and we replaced it with another relevant article (Cummins, 2009; Mehraliana, 2012; Bhaskaran, 2021; and Putri, 2020). We thank you for your input and comments.

6. There is no need to explain β_0 in page 10.

Thanks for the suggestion, we remove the β0 at line 335 and 396

7. The sections Result and Discussion and Result, should be Results and Discussion, and Results.

We separate the result and discussion section. For result, it explain about the data. And discussion, we combine between data that we got with theoretical or previous result which are in line to support the research finding.

8. Please remove tables 2, 5, 8, 11. They do not add anything to the academic discussion. They are just the data of the paper.

We follow your advise to delete this part.

9. In tables 1, 4, 7, 10, please remove T-ratio. You have already reported Standard Error. No need to report them both. Alternatively, you can drop standard error column, and keep T-ratio.

We delete the t-ratio following your suggestion. (table 2-6.

10. In tables 3, 6, 9 and 12, you do not have to report all those statistics under “Effects Specification” part. Adding one of the R-squares and number of observations should be enough.

Thanks for the advise. We follow your instruction. (Table 7-11)

11. The authors need to think about generalizability of these results. Can these findings be used for other countries? At what conditions? Maybe they can think in this study, or future studies if not possible here, to do a panel regression of all firms, in all countries, and they can control for macro level variables, or share of the Muslim population in the country, or maybe some variable where they measure observability of the religion.

It is possible for other country to use the same model to calculate the efficiency and for panel data model, future research can add the other controlling variables such as number of muslim population or other macroeconomics variable (Consumption, GDP). We explain this in line 839-851.

12. Please remove Supporting Info part of the paper. It is not relevant for an article at PlosONE.

We follow your suggestion to delete the support information and put another table (The List of company name) following the suggestion from 1^st^ reviewer.

Referenes

Bellemare, M. (2020) “How to Write Applied Papers in Economics” Working paper <http://marcfbellemare.com/wordpress/wp-content/uploads/2020/09/BellemareHowToPaperSeptember2020.pdf>

Joan C. Henderson, (2016) Halal food, certification and halal tourism: Insights from Malaysia and Singapore, Tourism Management Perspectives, Volume 19, Pages 160-164, ISSN 2211-9736, https://doi.org/10.1016/j.tmp.2015.12.006.

Secinaro, S. and Calandra, D. (2021), "Halal food: structured literature review and research agenda", British Food Journal, Vol. 123 No. 1, pp. 225-243. https://doi.org/10.1108/BFJ-03-2020-0234

Balli, Faruk, Billah, Mabruk, Hoxha, Indrit (forthcoming) “Extreme Connectedness of Agri-Commodities with Stock Markets and its Determinants”, Global Finance Journal, forthcoming.

6. PLOS authors have the option to publish the peer review history of their article ([what does this mean?](https://journals.plos.org/plosone/s/editorial-and-peer-review-process#loc-peer-review-history)). If published, this will include your full peer review and any attached files.

If you choose “no”, your identity will remain anonymous but your review may still be made public.

**Do you want your identity to be public for this peer review?** For information about this choice, including consent withdrawal, please see our [Privacy Policy](https://www.plos.org/privacy-policy).

Reviewer #1: No

Reviewer #2: No

[NOTE: If reviewer comments were submitted as an attachment file, they will be attached to this email and accessible via the submission site. Please log into your account, locate the manuscript record, and check for the action link "View Attachments". If this link does not appear, there are no attachment files.]

While revising your submission, please upload your figure files to the Preflight Analysis and Conversion Engine (PACE) digital diagnostic tool, <https://pacev2.apexcovantage.com/>. PACE helps ensure that figures meet PLOS requirements. To use PACE, you must first register as a user. Registration is free. Then, login and navigate to the UPLOAD tab, where you will find detailed instructions on how to use the tool. If you encounter any issues or have any questions when using PACE, please email PLOS at [figures@plos.org](mailto:figures@plos.org). Please note that Supporting Information files do not need this step.

*In compliance with data protection regulations, you may request that we remove your personal registration details at any time.* [*(Remove my information/details)*](https://www.editorialmanager.com/pone/login.asp?a=r)*. Please contact the publication office if you have any questions.*
